# Review of the status of neoadjuvant therapy in HER2-positive breast cancer

**DOI:** 10.3389/fonc.2023.1066007

**Published:** 2023-01-30

**Authors:** Gavin P. Dowling, Stephen Keelan, Sinead Toomey, Gordon R. Daly, Bryan T. Hennessy, Arnold D. K. Hill

**Affiliations:** ^1^ Medical Oncology Lab, Department of Molecular Medicine, Royal College of Surgeons in Ireland, Dublin, Ireland; ^2^ The Department of Surgery, Royal College of Surgeons in Ireland, Dublin, Ireland; ^3^ The Department of Surgery, Beaumont Hospital, Dublin, Ireland

**Keywords:** neoadjuvant therapy, breast cancer, HER2 (human epidermal growth factor 2), targeted therapy, biomarker, antibody-drug-conjugates

## Abstract

**Purpose:**

The development of human epidermal growth factor receptor 2 (HER2)-directed therapies has revolutionized the treatment of HER2-positive breast cancer. The aim of this article is to review the continually evolving treatment strategies in the neoadjuvant setting of HER2-positive breast cancer, as well as the current challenges and future perspectives.

**Methods:**

Searches were undertaken on PubMed and Clinicaltrials.gov for relevant publications and trials.

**Findings:**

The current standard of care in high-risk HER2-positive breast cancer is to combine chemotherapy with dual anti-HER2 therapy, for a synergistic anti-tumor effect. We discuss the pivotal trials which led to the adoption of this approach, as well as the benefit of these neoadjuvant strategies for guiding appropriate adjuvant therapy. De-escalation strategies are currently being investigated to avoid over treatment, and aim to safely reduce chemotherapy, while optimizing HER2-targeted therapies. The development and validation of a reliable biomarker is essential to enable these de-escalation strategies and personalization of treatment. In addition, promising novel therapies are currently being explored to further improve outcomes in HER2-positive breast cancer.

## Introduction

Human epidermal growth factor receptor 2 (HER2) is overexpressed and/or amplified in 15-20% of all breast cancers. Before the advent of HER2-directed therapies, this subtype was associated with an aggressive clinical course and poor outcomes (1, 2). The introduction of trastuzumab, the first humanized anti-HER2 monoclonal antibody, transformed the treatment of HER2-positive breast cancer. The benefit of neoadjuvant breast cancer treatment with chemotherapy, endocrine therapy and/or targeted therapy is well established to downstage disease, improve resectability and potentially reduce the extent of breast and axillary surgery (3–5). Specifically, in the HER2-positive subtype, additional benefits of neoadjuvant systemic therapy have been appreciated. These include the potential to individualize adjuvant therapy options based on pathological response and to provide information about tumor status *in vivo*, allowing for escalation or de-escalation of therapy, as guided by response biomarkers. Thus, the current standard of care in patients with high-risk HER2-positive breast cancer is a combination of chemotherapy combined with dual anti-HER2 therapy (6, 7). This review will discuss the evolving standard of care in the neoadjuvant setting of HER2-positive breast cancer, as well as the challenges and future perspectives.

## Review of neoadjuvant trials

Neoadjuvant therapy is the current standard of care for treating ≥T2 or node-positive HER2-positive breast cancer. Pathological complete response (pCR) is most commonly defined as the absence of residual invasive cancer of the complete resected breast specimen and all sampled regional lymph nodes following completion of neoadjuvant systemic therapy (ypT0/Tis ypN0) (8). pCR at surgery is correlated with favorable patient outcomes, particularly in HER2-positive, hormone-receptor (HR)-negative breast cancer, as demonstrated by the CTNeoBC pooled analysis. This meta-analysis, performed by the FDA, included 11,955 patients across 12 neoadjuvant trials, with a minimum follow-up of 3 years, to evaluate pCR as a surrogate endpoint for improved long-term outcomes in breast cancer. Across all subgroups, pCR was associated with improved event-free survival (EFS) (HR 0.48; 95% CI 0.43-0.54) and overall survival (OS) (HR 0.36; 95% CI 0.31-0.42). Three trials were included for HER2-positive breast cancer: NOAH, TECHNO and GeparQuattro (9). Several additional meta-analyses have since supported the value of pCR as an informative surrogate biomarker for enhanced survival in HER2-positive breast cancer (10–12).

Trastuzumab is a monoclonal antibody against HER2 that binds to an extracellular domain of this receptor and prevents ligand-independent HER2-mediated signaling (13). Following its success in treating advanced and early-stage HER2-positive disease, multiple neoadjuvant trials that combine chemotherapy with trastuzumab have been performed. The NOAH trial, for example, reported that the addition of trastuzumab to neoadjuvant chemotherapy had a significant improvement on both pCR and EFS when compared to chemotherapy alone (14). Similar improvements in pCR were seen with the addition of trastuzumab to a chemotherapy backbone in the TECHNO trial and GeparQuattro study (15, 16).

Pertuzumab binds to the extracellular domain II of HER2, which results in ligand-dependent HER2–HER3 dimerization (17). This mechanism of action is complementary to that of trastuzumab. In the NeoSphere trail, a pCR rate of approximately 45% was observed in patients treated with pertuzumab plus trastuzumab and docetaxel, compared to those who received only trastuzumab and docetaxel (29%) (35). This combination of pertuzumab with trastuzumab and docetaxel was also investigated in the CLEOPATRA trial, which reported a significant overall survival benefit (56.5 months vs 40.8 months) (18). Following these trials, dual HER2-blockade with trastuzumab and pertuzumab in combination with standard neoadjuvant chemotherapy became the standard of care (7, 19).

Lapatinib is a dual reversible tyrosine kinase inhibitor that selectively targets and inhibits HER2 and epidermal growth factor receptor (EGFR) (20). Lapatinib has demonstrated activity in HER2-positive metastatic breast cancer that had progressed on trastuzumab-containing therapy (21). In the Cher-LOB trial, patients treated with lapatinib and trastuzumab plus chemotherapy showed a relative 80% increase in pCR rate, compared to treatment with either trastuzumab or lapatinib plus chemotherapy (22). Additionally, the CALGB 40601 trial showed improved 7-year relapse-free survival and OS (23). Despite several studies showing improved pCR rates with the addition of lapatinib to trastuzumab and chemotherapy in the neoadjuvant setting, these long-term outcomes have not been consistent across trials (24–27). The inconsistency of long-term outcomes, along with the less favorable adverse event profile associated with the addition of lapatinib, has prevented it from becoming a currently recommended neoadjuvant treatment.

Both anthracycline and non-anthracycline-based chemotherapy regimens are well established as neoadjuvant treatments of HER2-positive breast cancer. Combination treatment with anthracyclines and trastuzumab can have significant side effects in patients, including febrile neutropenia and cardiotoxicity. Multiple trials have explored the feasibility of treating these patients with anthracycline-free regimes. The TRAIN-2 trial reported high pCR rates after neoadjuvant chemotherapy with or without anthracyclines plus dual-HER2 blockade. No significant difference was seen in either pCR or patient outcomes between the two groups (28). In addition, the TRYPHAENA trial showed similar efficacy for anthracycline-free compared to anthracycline-containing regimens together with standard anti-HER2 therapy. Cardiac safety was the primary endpoint: left ventricular systolic dysfunction (LVSD) incidence was low (5.6%) in the neoadjuvant setting in the anthracycline-containing arm (29). Furthermore, the BERENICE trial demonstrated cardiac safety in both dose-dense and standard anthracycline-containing regimens in combination with trastuzumab and pertuzumab (30, 31).

Given the success of neoadjuvant systemic chemotherapy with dual HER2-blockade, achieving pCR rates of up to 65% in some studies (28, 29), the possibility of replacing chemotherapy with an agent associated with less toxicity was explored. The phase III KRISTINE study compared neoadjuvant trastuzumab emtansine (T-DM1), an antibody-drug conjugate, plus pertuzumab, with conventional systemic chemotherapy plus dual HER2-blockade. The results showed that the proportion of patients who achieved pCR was significantly greater in patients receiving traditional neoadjuvant chemotherapy plus trastuzumab and pertuzumab than those who received T-DM1 plus pertuzumab (56% vs 44%) (32). The results of these neoadjuvant clinical trials are summarised in **Table 1**.

**Table 1:** Neoadjuvant Trials in HER2-positive breast cancer.

Trial	Phase	Treatment	Treatment arms	Survival	N	pCR rate
Trastuzumab						
NOAH (14)	III	Trastuzumab	DTCMF + H	EFS (3y) 71% OS (3y) 87%	117	38%
			DTCMF	EFS (3y) 56% OS (3y) 79%	118	19%
GeparQuattro (15)	III	Trastuzumab	H + EC → H + Dc		146	33%
			H + EC → H + DcCp		47	31%
			H + EC → H + Dc → H + Cp		136	35%
TECHNO (16)	II	Trastuzumab	EC → HT	DFS (3y) 77.9% OS (3y) 89.4%	217	39%
Trastuzumab + Lapatinib						
NeoALTTO (24)	III	Lapatinib + Trastuzumab	LH → HT	EFS (6y) 74% OS (6y) 85%	68	47%
			H → HT	EFS (6y) 67% OS (6y) 82%	40	28%
			L → HT	EFS (6y) 67% OS (6y) 79%	30	20%
CHER-LOB (22)	II	Lapatinib + Trastuzumab	LH → TFEC + LH	RFS (5y) 86%	46	47%
			H → TFEC + H	RFS (5y) 78%	36	25%
			L → TFEC + L	RFS (5y) 77%	38	26%
CALGB 40601 (23)	III	Lapatinib + Trastuzumab	THL	RFS (7y) 93% OS (7y) 96%	118	57%
			TH	RFS (7y) 79% OS (7y) 88%	120	45%
			TL	RFS (7y) 69% OS (7y) 84%	67	30%
NSABP B-41 (33)	III	Trastuzumab + Lapatinib	DC + H → T + H	DFS (5y) 84.3% OS (5y) 94.5%	177	49.4%
			DC + L → T + H	DFS (5y) 78.6% OS (5y) 89.4%	159	47.4%
			DC + LH → T + H	DFS (5y) 90% OS (5y) 95.7%	165	60.2%
Lapatinib						
GeparQuinto (34)	III	Lapatinib	ECH → Dc + H	DFS (3y) 84.8% OS (3y) 91.7%	307	30.3%
			ECL → Dc + L	DFS (3y) 83.7% OS (3y) 93.6%	308	22.7%
Trastuzumab + Pertuzumab						
NeoSphere (35)	II	Pertuzumab + Trastuzumab	H + Dc	PFS (5y) 81% DFS (5y) 81%	107	29%
			HP + Dc	PFS (5y) 86% DFS (5y) 84%	107	46%
			HP	PFS (5y) 73% DFS (5y) 80%	107	17%
			P + Dc	PFS (5y) 73% DFS (5y) 75%	96	24%
TRYPHAENA (29)	II	Pertuzumab + Trastuzumab	FEC + HP → Dc + HP	DFS (3y) 87% PFS (3y) 89% OS (3y) 94%	73	62%
			FEC → Dc + HP	DFS (3y) 88% PFS (3y) 89% OS (3y) 94%	75	57%
			Dc + Cb + HP	DFS (3y) 90% PFS (3y) 87% OS (3y) 93%	77	66%
GeparSepto (36)	III	Pertuzumab + Trastuzumab	T + HP → EC + HP	iDFS (4y) 89%	199	54%
			Nab-T + HP → EC + HP		197	62%
ADAPT HER2+/HR- (37)	II	Pertuzumab + Trastuzumab	HP	iDFS (5y) 87% OS (5y) 94%	92	34%
			HP + T	iDFS (5y) 98% OS (5y) 98%	42	90%
BERENICE (30, 38)	II	Pertuzumab + Trastuzumab	dd D + C → T + HP	EFS (5y) 91% OS (5y) 96%	199	62%
			FEC → Dc + HP	EFS (5y) 89% OS (5y) 94%	201	61%
TRAIN-2 (28)	III	Pertuzumab + Trastuzumab	FEC (x3) → TCb + HP (x6)	EFS (3y) 93% OS (3y) 98%	211	67%
			TCb + HP (x9)	EFS (3y) 94% OS (3y) 98%	206	68%
PHERGain (39)	II	Pertuzumab + Trastuzumab	DcCb + HP		71	58%
			HP		285	35%
PREDIX HER2 (40)	II	Pertuzumab + Trastuzumab	Dc + HP		99	46%
T-DM1 + Pertuzumab						
KRISTINE (32)	III	T-DM1 + Pertuzumab	T-DM1 + P	EFS (3y) 85% iDFS (3y) 93%	223	44%
			DcCb + HP	EFS (3y) 94% iDFS (3y) 92%	221	56%

H- trastuzumab; P- pertuzumab; T- paclitaxel; D- doxorubicin; C- cyclophosphamide; M- methotrexate; F- fluorouracil; L- lapatinib; E- epirubicin; Dc- docetaxel; Cp- capecitabine; Cb- carboplatin, Nab-T- nab-paclitaxel; dd- dose dense; T-DM1- trastuzumab emtansine.

To prevent the potential of over-treatment in patients with low-risk HER2-positive breast cancer, the APT trial was designed. This study included patients with ≤3cm, node-negative, HER2-positive tumors. This trial showed excellent outcomes with adjuvant paclitaxel for 12 weeks plus 12 months of trastuzumab, with a 3-year IDFS of 98.7% and 7-year IDFS of 93% (41). Thus, primary surgery combined with adjuvant therapy should be offered to these patients, providing an effective de-escalated treatment regime.

## Adjuvant therapy in the context of neoadjuvant strategy/according to pCR status

Following completion of neoadjuvant therapy, subsequent adjuvant therapies can be guided by pCR status after surgery.

In patients who achieve pCR, current guidelines recommend continuing trastuzumab to complete a total of 12 months of anti-HER2 therapy (6, 42). Patients with initially node-positive disease should also continue pertuzumab for the remainder of the year, based on the findings of the adjuvant APHINITY trial. This trial concluded that the addition of pertuzumab in the adjuvant setting may significantly improve invasive disease-free survival in patients with node-positive disease (43). However, no statistically significant difference was seen in OS after a median follow-up of 8.4 years (44).

In patients who do not achieve pCR, adjuvant therapy with T-DM1 should be offered instead of trastuzumab monotherapy. This recommendation is based on the results from the KATHERINE trial. In this phase III trial, patients with residual invasive tumors after neoadjuvant therapy were randomly assigned to received either adjuvant T-DM1 or trastuzumab for 14 cycles. Treatment with T-DM1 significantly improved invasive disease-free-survival (iDFS) compared to treatment with trastuzumab (88.3% vs. 77.0%, respectively, HR 0.50, 95% CI, 0.39–0.64; *p* < 0.001) (45).

Adjuvant treatment with T-DM1 in stage I HER2-positive breast cancer was investigated in the ATEMPT trial. This trial aimed to establish if adjuvant T-DM1 would be associated with less toxicity than paclitaxel plus trastuzumab without compromising invasive disease-free-survival (iDFS). Although one year of T-DM1 had a 3-year iDFS of 97.8%, T-DM1 failed to demonstrate reduced toxicity compared to paclitaxel and trastuzumab (46).

The KAITLIN study was another trial which aimed to replace taxanes and trastuzumab with T-DM1. In this trial, patients with node-positive or high-risk node-negative (HR negative and tumor size >2cm) HER2-positive breast cancer were randomly assigned to anthracycline chemotherapy followed by trastuzumab and a taxane plus pertuzumab or anthracycline chemotherapy followed by T-DM1 plus pertuzumab. The results showed no significant difference in 3-year iDFS rate between the two arms of the study (47).

## Duration of anti-HER2 therapy

The current standard of care is to complete 12 months of anti-HER2 therapy. The benefit of this therapy was demonstrated in the crucial HERA, NCCTG N9831, NSABP B-31 and BCIRG-006 trials. It was shown that adjuvant trastuzumab with standard chemotherapy reduced the relative risk of death by up to 30% and the relative risk of recurrence by up to 40% (48–52).

The HERA trial demonstrated that a longer duration of the same anti-HER2 therapy did not improve efficacy, in which two years was compared to one year of trastuzumab treatment. In those who received two years of therapy, no additional benefit in disease free survival (DFS) was seen and associated with a higher rate of cardiotoxicity (48).

Given the effectiveness of anti-HER2 therapy, multiple trials were designed to evaluate the efficacy of reduced duration of treatment.

The PHARE, HORG and PERSEPHONE trials compared 6 months to 12 months of trastuzumab treatment (53–55). PERSEPHONE is the only trial to date to have reached its non-inferiority endpoint. In this trial of 4089 patients, after a median follow-up of 5.4 years, those assigned to 6 months of trastuzumab therapy experienced non-inferior 4-year DFS rates compared to those receiving 12 months (89.4 versus 89.8 percent, respectively; HR 1.07, 95% CI 0.93-1.24), with less cardiotoxicity leading to discontinuation of trastuzumab (55).

A shortened course of 9-weeks of trastuzumab therapy was evaluated in the SOLD and ShortHER trials. These trials failed to reach the non-inferiority endpoint for DFS (56, 57). Despite being unable to claim non-inferiority, recently presented follow-up data of the ShortHER trial confirmed favorable long-term outcomes in terms of OS and DFS with a 9 week course of trastuzumab (58). The results of these trials are summarised in **Table 2**.

**Table 2:** Trials investigating the duration of anti-HER2 therapy.

Trial	Phase	Duration of trastuzumab	N	Efficacy versus 1-year trastuzumab	Cardiac events
HERA (48)	III	2 years	5099	DFS (10y) 70% vs 72%	7.3% vs 4.4%
PHARE (53)	III	6 months	3380	DFS (3y) 87.7% vs 90.7%	1.9% vs 5.7%
HORG (54)	III	6 months	481	DFS (3y) 93.3% vs 95.7%	0.8% vs 0%
PERSEPHONE (55)	III	6 months	4088	DFS (4y) 89.4% vs 89.8%	8% vs 11%
SOLD (56)	III	9 weeks	2174	DFS (5y) 88% vs 90.5%	2% vs 4%
ShortHER (58)	III	9 weeks	1253	DFS (5y) 85% vs 88%	4.3% vs 13.1%

A patient-level meta-analysis of 5 trials investigating shorter adjuvant trastuzumab treatment found that 6 months of treatment with trastuzumab is non-inferior to 12 months, but 9 weeks is not (59).

Escalation of adjuvant anti-HER2 therapy has also been evaluated in patients with higher-risk disease. As previously discussed, the APHINITY trial showed that patients with HER2-positive, node-positive disease benefited from the addition of pertuzumab to trastuzumab in the adjuvant setting (3-year iDFS of 92% vs 90.2%, HR 0.77; 95% CI, 0.62 to 0.96; P=0.02) (43). Extended adjuvant therapy with neratinib, a tyrosine kinase inhibitor, after trastuzumab therapy was investigated in the phase 3 ExteNet trial. This trial showed a benefit in 5-year iDFS of 90.2% in patients receiving neratinib, compared with 87.7% of those receiving the placebo (HR 0.73, 95% CI 0.57–0.92). Subgroup analysis revealed that in patients with HR-positive cancer a benefit of 5.1% in iDFS (HR 0.58, 95% CI 0.41–0.82) was shown. However, as patients in the ExteNET trial had neither received pertuzumab nor T-DM1, the actual benefit after current adjuvant and post-neoadjuvant targeted therapy could be smaller (60). Nevertheless, neratinib could offer an additional treatment option in patients with HR-positive disease.

## De-escalation strategies

The possibility of further therapy de-escalation in low-risk disease is currently being investigated in multiple clinical trials.

The WSG ADAPT HER+/HR- trial explored the feasibility of de-escalated neoadjuvant therapy in 134 patients with HER2-positive, HR-negative disease. In this trial, patients were randomly assigned to receive trastuzumab plus pertuzumab, either with or without paclitaxel. Remarkably high pCR rates (90.5%) were reported in the de-escalated chemotherapy arm after 12 weeks of paclitaxel plus dual HER2 blockade. Adjuvant therapy was given as per national guidelines. Interestingly, adjuvant chemotherapy could be omitted in patients achieving pCR at the physician’s discretion, and 79% of patients who achieved pCR in the paclitaxel arm received no further chemotherapy (61). In May 2022, survival outcomes from the trial were published. Notably, patients who achieved pCR had a 5-year iDFS rate of 98%, regardless of whether they received neoadjuvant paclitaxel or were in the chemotherapy-free arm (37). While this trial is not powered to prove non-inferiority of a chemotherapy-sparing approach, these results pave the way for larger randomized control trials designed to specifically investigate whether the omission of chemotherapy may be feasible in carefully pre-selected patients.

The trial of patients with HR-positive disease, ADAPT-TP HER2+/HR+, found that patients given neoadjuvant T-DM1 alone or with endocrine therapy were significantly more likely to achieve pCR than those given trastuzumab with endocrine therapy (41%/41.5% vs 15.1%, p<0.001). Survival data from ADAPT-TP revealed that patients who achieved pCR had similar 5-year DFS rates, regardless of whether they received chemotherapy (92.1% (95%-CI: 78-97%) with adjuvant chemotherapy vs 93% (84-97%) without adjuvant chemotherapy) (62).

As a result of these trials, further de-escalation trials were designed to prevent over-treatment. The CompassHER2-pCR and DESCRESCENDO trials are ongoing and aim to individualize adjuvant therapy based on pCR status after a de-escalated neoadjuvant course of 12 weeks paclitaxel with trastuzumab plus pertuzumab (63, 64). ATEMPT 2.0 is a phase 2 trial comparing adjuvant T-DM1 followed by trastuzumab to paclitaxel and trastuzumab, followed by trastuzumab alone. It aims to evaluate where the T-DM1 arm will have less toxicity and improved outcomes (65).

Furthermore, the omission of surgery is currently being investigated in low-risk HER2-positive early breast cancer patients who achieve a complete response to neoadjuvant therapy. In the ELPIS trial, if a complete response of the tumor is reported on the post-neoadjuvant therapy breast MRI, a vacuum-assisted breast biopsy (VAC) is performed. If on VAC no invasive or *in situ* disease is found, patients will be eligible to omit loco-regional surgery. They will instead proceed to have whole breast radiotherapy and complete 1 year of trastuzumab and pertuzumab (66).

## Biomarkers

The next challenge to enable further individualization of neoadjuvant treatment in HER2-positive breast cancer is the development of a robust biomarker to predict pCR. This would allow for the adjustment of neoadjuvant therapy by identifying patients with an increased likelihood of achieving pCR based on favorable predictive biomarkers, and identifying patients with an exceptional response to neoadjuvant therapy, who may be candidates for the omission of surgery altogether. To date, no biomarker has been validated and current recommendations are that biomarkers should not be used for monitoring patients receiving neoadjuvant therapy (67). Further research is ongoing to develop and validate potential biomarkers.

HER2-enriched intrinsic subtype, a tissue-based biomarker, has been linked with high pCR rates following neoadjuvant therapy (68). Retrospective analyses of the NOAH (69), NeoALTTO (70), CALGB40601 (71) and CHER-LOB (72) trials reported the HER2-enriched subtype to have an increased likelihood of achieving pCR with neoadjuvant chemotherapy and anti-HER2 therapy compared to other subtypes. A combined analysis of the PAMELA and TBCRC006/023 trials demonstrated that combining HER2-enriched subtype and ERBB2 mRNA levels has better sensitivity than each variable alone in predicting pCR in chemotherapy-sparing regimens (73).

Several studies have investigated tumor-infiltrating lymphocytes (TILs) as another potential biomarker for the prediction of pCR following neoadjuvant therapy in HER2-positive breast cancer. One meta-analysis reported that, regardless of the anti-HER2 agents and chemotherapy used, higher baseline TILs were associated with increased likelihood of achieving pCR (74). The PAMELA trial investigated the association between TILs and pCR in patients treated with trastuzumab and lapatinib. This study found that the presence of on-treatment TILs in HER2-positive breast cancer, measured on day 15 of treatment, was significantly associated with pCR (75). Further studies are needed to validate TILs as an accurate biomarker before it can be considered for use in clinical practice.

A pooled analysis of five prospective trials reported that PIK3CA mutant tumors significantly decreased pCR rates in HER2-positive breast cancer, particularly in HR-positive tumors (76). However, biomarker analysis of the NeoSphere study reported a non-significant decrease in pCR in patients with mutated PIK3CA (77). Therefore, PIK3CA warrants further investigation before it can be considered a potential biomarker for predicting pCR in these patients.

Blood-based biomarkers, such as circulating tumor cells (CTCs) and circulating tumor DNA (ctDNA), have also been investigated as potential predictors of pCR. One meta-analysis reported that detection of CTCs before starting neoadjuvant therapy for breast cancer was associated with a slightly lower rate of pCR (78), however, further evidence is needed to validate this. A sub-study of the NeoALTTO trial found that ctDNA detection before neoadjuvant anti-HER2 therapy was associated with decreased pCR rates (79). ctDNA detection after completion of neoadjuvant therapy has also been shown to be associated with residual disease (80–82).

Lastly, imaging-based biomarkers are also being explored as predictors of response to treatment. The use of fluorodeoxyglucose positron emission tomography (FDG-PET) as a biomarker was evaluated in the NeoALTTO (83), PHERGain (39) and TBCRC026 (84) trials. These studies suggest that these imaging strategies could facilitate further tailoring of therapy, although such strategies will require additional clinical investigation.

## Future perspectives

With substantially improved outcomes associated with the development of HER2-targeted therapies in recent years, several novel HER2-directed agents are currently being investigated in clinical trials, with promising results.

## Trastuzumab deruxtecan

Trastuzumab deruxtecan (T-DXd) is an antibody-drug conjugate, which is composed of a monoclonal antibody targeting HER2, a cleavable tetrapeptide-based linker and a topoisomerase I inhibitor (85). It has a significantly higher drug-to-antibody ratio than other antibody-drug conjugates, however the stability of the linker seems to allow for high efficacy without significant side effects. The cytotoxic payload, deruxtecan, is cell membrane permeable, giving the drug its bystander-killing effect (86).

T-DXd has shown promising results in HER2-positive breast cancer patients in the metastatic setting. In the DESTINY-Breast 01 trial, T-DXd showed a substantial benefit in patients with HER2-positive metastatic breast cancer who had previously received treatment with T-DM1 (87). Significantly improved overall response rate (ORR) and progression-free survival (PFS) was reported with T-DXd compared to T-DM1 in HER2-positive metastatic breast cancer treated with trastuzumab and a taxane in the DESTINY-Breast 03 trial (88). More recently, in the DESTINY-Breast 04 trial involving patients with HER2-low metastatic breast cancer, treatment with T-DXd resulted in significantly longer PFS and OS than the physician’s choice of chemotherapy (89).

Given the promising results of T-DXd in HER2-positive breast cancer in the metastatic setting, adjuvant and neoadjuvant T-DXd is currently under investigation. The ongoing DESTINY-Breast 05 trial is investigating T-DXd in high-risk HER2-positive disease with residual invasive breast cancer following neo-adjuvant therapy, compared to T-DM1 (90). Neoadjuvant T-DXd is also being evaluated in locally advanced or inflammatory HER2-positive breast cancer patients in the ongoing DESTINY-Breast 11 trial. This trial will compare T-DXd, alone or followed by docetaxel, trastuzumab and pertuzumab, to the current standard of care regimen (ddAC-THP) (91). The SHAMROCK study is another trial of neoadjuvant T-DXd in early stage HER2-positive breast cancer, which incorporates therapy escalation and de-escalation strategies using an on-treatment biopsy and imaging (92).

## Other novel agents

Tucatinib, a potent and selective tyrosine kinase inhibitor of HER2, is another promising agent. Tucatinib was added to trastuzumab and capecitabine in the HER2CLIMB study, resulting in improved PFS and OS in heavily pre-treated metastatic HER2-positive breast cancer (93). These results led to the design of the HER2CLIMB-05 trial, which will investigate the addition of tucatinib to standard of care maintenance in the first line setting for patients with HER2-positive metastatic breast cancer (94). Adjuvant tucatinib, in combination with T-DM1, is currently being evaluated in patients with residual disease following neo-adjuvant therapy in the COMPASS HER2 RD trial (95).

Several immune checkpoint inhibitors have been investigated in combination with HER2-directed therapies in patients with metastatic disease. Subgroup analyses from the PANACEA trial, which investigated treatment with pembrolizumab and trastuzumab in patients who had progressed on trastuzumab, showed that higher response rates were seen in PD-L1 positive tumors (96). Similarly, the KATE2 trial observed favorable PFS with atezolizumab in the subgroup of patients with PD-L1 positive tumors (97). Atezolizumab, a PD-L1 inhibitor, is being evaluated in the adjuvant setting in combination with T-DM1 in patients with residual disease after neoadjuvant therapy (98). Recently, neoadjuvant atezolizumab was investigated with docetaxel, trastuzumab and pertuzumab in HER2-positive early breast cancer and reported an acceptable pCR rate and modest toxic effects (99). Further trials on neoadjuvant immunotherapy in early HER2-positive breast cancer underway, such as NeoHIP (100) and APTneo (101) studies are underway.

## Conclusion

The introduction of HER2-directed therapies perioperatively has revolutionized the treatment of patients with HER2-positive early breast cancer. Neoadjuvant chemotherapy in combination with trastuzumab and pertuzumab has led to increased pCR rates, which in turn has significantly improved outcomes in these patients. Pathological response status provides an important guide for the appropriate adjuvant systemic therapy. De-escalation strategies are currently being investigated to avoid over treatment, and aim to safely reduce chemotherapy, while optimizing HER2-targeted therapies. The development and validation of a reliable biomarker is essential to enable these de-escalation strategies and personalization of treatment. In addition, promising novel therapies are currently being explored to further improve outcomes in HER2-positive breast cancer.

## Author contributions

Conceptualisation – AH, BH, GPD. Literature research – GPD. Manuscript preparation – GPD and SK. Manuscript review – GPD, SK, ST, GRD, BH, and AH. All authors contributed to the article and approved the submitted version.
